# Long-term results on the suppression of secondary brain injury by early administered low-dose baclofen in a traumatic brain injury mouse model

**DOI:** 10.1038/s41598-023-45600-7

**Published:** 2023-10-30

**Authors:** Ji Young Park, Junwon Park, Jiwon Baek, Jin Woo Chang, Young Goo Kim, Won Seok Chang

**Affiliations:** 1 Department of Neurosurgery, Yonsei University College of Medicine, 50 Yonsei-ro, Seodaemun-gu, Seoul, 03722 Republic of Korea; 2 Department of Neurosurgery, Ewha Womans University School of Medicine, Ewha Womans University Mokdong Hospital, Mok 5-dong, Yangcheon-gu, Seoul, 07985 Republic of Korea; 3 Brain Korea 21 PLUS Project for Medical Science and Brain Research Institute, Yonsei University College of Medicine, Seoul, Republic of Korea

**Keywords:** Neuroscience, Neurology

## Abstract

Secondary injury from traumatic brain injury (TBI) perpetuates cerebral damages through varied ways. Attenuating neuroinflammation, which is a key feature of TBI, is important for long-term prognosis of its patients. Baclofen, a muscle relaxant, has shown promise in reducing excessive inflammation in other neurologic disorders. However, its effectiveness in TBI remains ambiguous. Thus, our study aimed to investigate whether early administration of baclofen could elicit potential therapeutic effects by diminishing exaggerated neuroinflammation in TBI mice. In this study, 80 C57BL/6 mice were used, of which 69 mice received controlled cortical impact. The mice were divided into six groups (11–16 mice each). Baclofen, administered at dose of 0.05, 0.2 and 1 mg/kg, was injected intraperitoneally a day after TBI for 3 consecutive weeks. 3 weeks after completing the treatments, the mice were assessed histologically. The results showed that mice treated with baclofen exhibited a significantly lower volume of lesion tissue than TBI mice with normal saline. Baclofen also reduced activated glial cells with neurotoxic immune molecules and inhibited apoptotic cells. Significant recovery was observed and sustained for 6 weeks at the 0.2 mg/kg dose in the modified neurological severity score. Furthermore, memory impairment was recovered with low-doses of baclofen in the Y-maze. Our findings demonstrate that early administration of low dose baclofen can regulate neuroinflammation, prevent cell death, and improve TBI motor and cognitive abnormalities.

## Introduction

Traumatic brain injury (TBI) is a major cause of disability and death globally and a significant risk factor for diverse neurodegenerative diseases. TBI is typically classified into primary and secondary injuries. The primary impact immediately inflicts mechanical disruption to the lesion, whereas secondary injuries, such as ischemia, excitotoxicity, apoptosis, and inflammation, contribute to neurodegeneration in multifactorial ways following primary insult^1^. Since secondary injuries are critical in determining the severity of the injury, numerous studies have focused on preventing secondary injury in the brain^2,3^.

Among the various therapeutic approaches, reducing neuroinflammation is a key target for TBI^4,5^. The activation of innate immune cells, including microglia, astrocytes, and oligodendrocytes, stands for the first stage of the inflammatory system in the central nervous system (CNS). This acute response protects the brain tissue from cell debris by releasing anti-inflammatory cytokines; however, the immoderate and continuous reaction in the chronic phase exacerbates the perilesional tissue through the neurotoxic materials^6^. The upregulated active form of glial cells with increased proinflammatory cytokines in this period eventually triggers complement-mediated activation and neuronal cell death^7^. This acceleration of secondary injury by neuroinflammation may result in behavioral abnormalities and cognitive dysfunction in TBI. Therefore, inhibition of the persistent inflammatory response might be a preventive and advantageous strategy for patients with TBI^8,9^.

There have been many drug candidates targeting the attenuation of neuroinflammation from preclinical and clinical trials, but significant progress in their clinical application has not been achieved yet^10^. Baclofen, a viable GABA-B receptor agonist approved by FDA, is usually used to alleviate spasticity, that can occur in patients with TBI, as well as cerebral palsy, stroke, or other motor dysfunction diseases^11,12^. Several recent clinical reports have reported that baclofen administration leads to an unanticipated but marked improvement in functional damage and consciousness with anti-spastic effects in TBI or non-TBI patients^13–15^. In this regard, there have been various assumptions to prove the partial role of GABA-B; however, no apparent reason has been elucidated. Several preclinical studies have proposed that GABA-B receptors are an important site of action for neuroinflammatory signals in in vitro and in vivo models^16–19^. Baclofen reduced proinflammatory cytokines in glial cells derived from multiple sclerosis^20^, recovered memory impairment, and attenuated astroglial activation at low doses in the cortex and hippocampus of a Streptozotocin-induced Alzheimer’s disease (AD) model^21^. However, little is known about the effects of baclofen on TBI-induced neuroinflammation.

Based on previous studies, we hypothesized that baclofen administration in the recovery phase might downregulate hyperactivated glial cells with neurotoxic molecules and improve neurological impairment. We investigated the possibility of early baclofen application to treat secondary injury of TBI by comparing three doses relevant to clinical cases.

## Results

### Baclofen prevented the loss of tissue volume of the lesion area in TBI mice brain.

**Figure 1:**
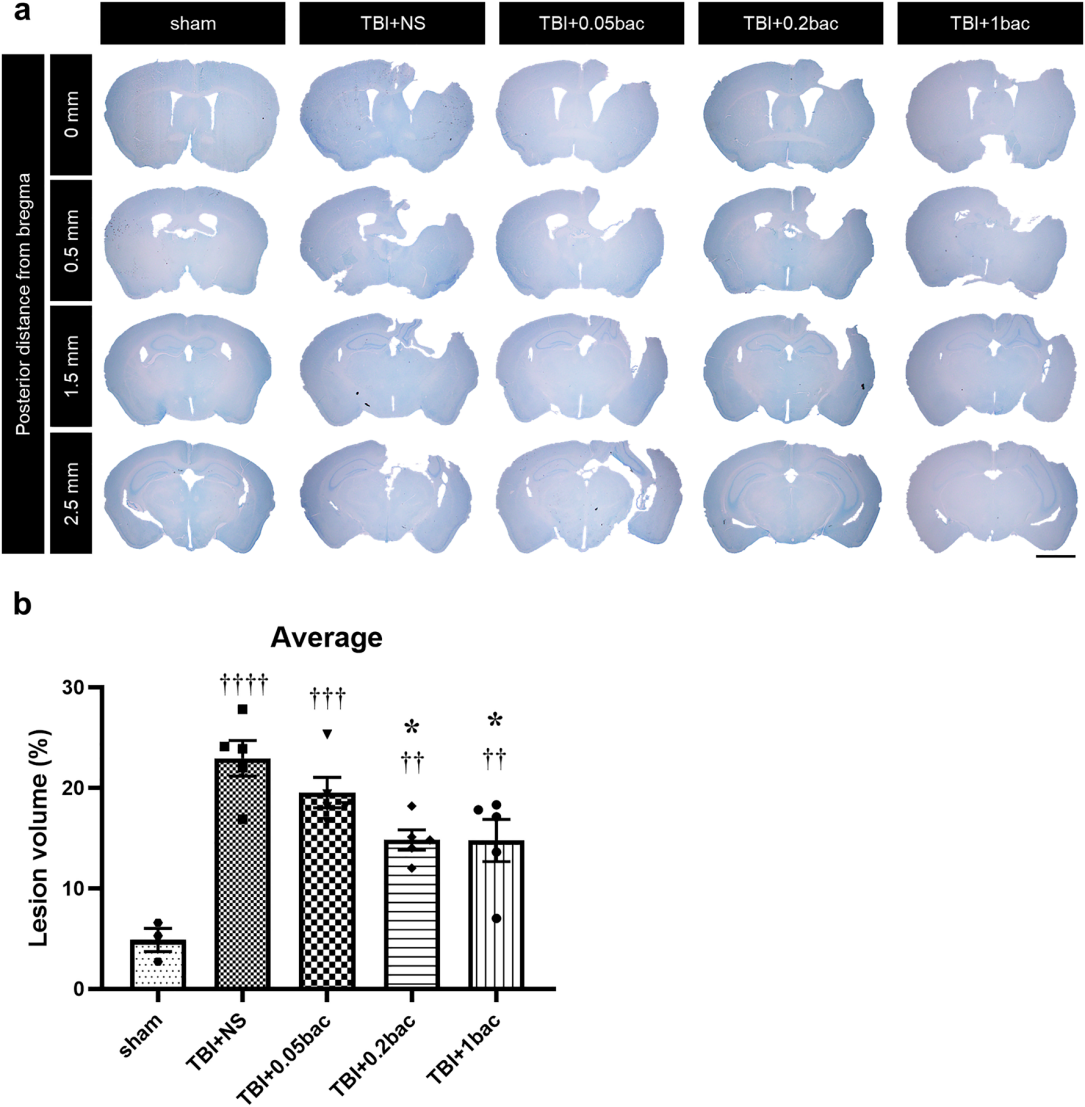
Baclofen inhibited loss of lesion volume of cortex region post-TBI. (**a**) Representative images of toluidine blue staining. Scale bar: 2 mm (**b**) Quantitative measurement of average lesion volume including 4 different distances from bregma. All data are represented as the mean ± SEM (n = 3–5). One-way ANOVA with Tukey’s multiple comparison test was performed. ††* p* < 0.01, †††* p* < 0.001, ††††* p* < 0.0001; compare with sham. *P < 0.05; compared with TBI + NS.

TBI mice model had extensive loss of their cortex and hippocampus region. We evaluated the proportion of lesions contrasted with the contralateral cortex at 6 weeks after TBI (Fig. 1a). The size of the lesion in all mice that underwent TBI modeling was significantly more extensive compared to the sham group (Fig. 1b, sham; 4.87 ± 1.15, TBI + normal saline (NS); 22.94 ± 1.78, *p* < 0.0001, TBI + 0.05 baclofen (bac); 19.52 ± 1.51, *p* = 0.0002, TBI + 0.2 bac; 14.82 ± 1, *p* = 0.0091, TBI + 1 bac; 14.76 ± 2.11, *p* = 0.0096 compared to the sham). However, in comparison to the TBI group, in the TBI + 0.2 bac (*p* = 0.0146) and TBI + 1bac groups (*p* = 0.0138), there was a lesser extent of tissue lose size compared with TBI + NS. In contrast, there was no significant difference in the TBI + 0.05 bac group.

### Baclofen decreased activated microglia and IL-1β level in the peri-lesion cortex following TBI

**Figure 2:**
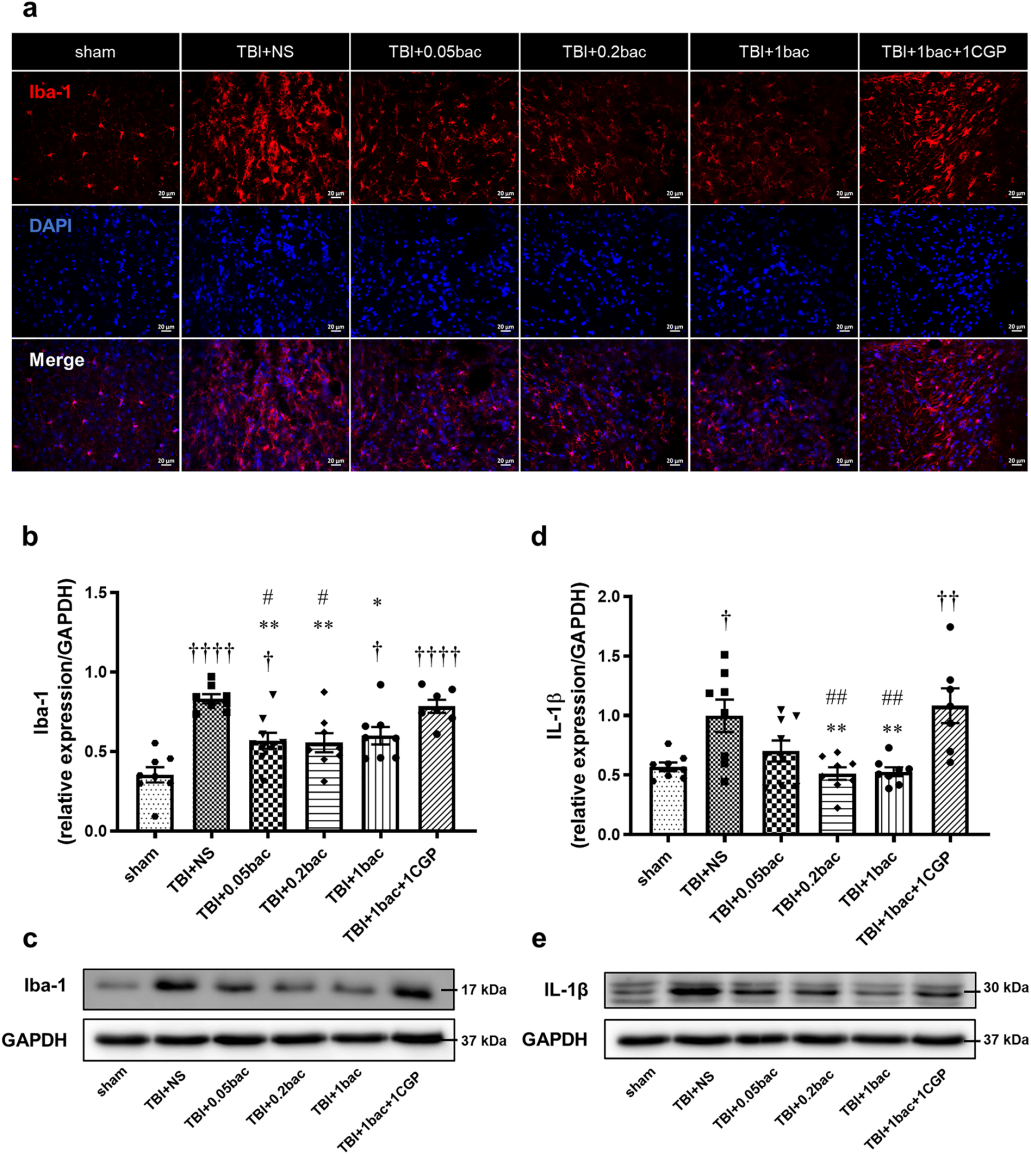
Early baclofen administration attenuated microglia and IL-1β in TBI. (**a**) Representative IF images of Iba-1 (red) and DAPI (blue) in the perilesional cortex area, 6 weeks after TBI. Scale bar: 20 μm. (**b**, **c**) Western blot result for Iba-1 showed the bar graph normalized relative to GAPDH and band intensity image for each group. (**d**, **e**) Quantitative analysis of western blot for IL-1β was also performed. All data are represented as the mean ± SEM (n = 7–9). One-way ANOVA with Tukey’s multiple comparison test was performed. † *p* < 0.05, †††† *p* < 0.0001; compared with sham. * *p* < 0.05, ** *p* < 0.01 compared with TBI + NS. # *p* < 0.05; compared with TBI + 1 bac + 1 CGP.

In this study, histological changes were verified in the peripheral cortex containing a lesion. Immunofluorescence data showed Iba-1, a representative marker of microglia, at 6 weeks after TBI (Fig. 2a). Western blot analysis was performed to confirm Iba-1 protein level activation (Fig. 2b,c). As a result, we observed a significant increase in Iba-1 expression levels in all TBI-induced groups compared with in the sham group, except for the group receiving 0.2 mg/kg of baclofen (sham; 0.35 ± 0.05: TBI + NS: 0.83 ± 0.03, *p* < 0.0001; TBI + 0.05 bac: 0.57 ± 0.05, *p* = 0.03; TBI + 0.2 bac: 0.56 ± 0.06, *p* = 0.058; TBI + 1 bac: 0.6 ± 0.06, *p* = 0.012; TBI + 1 bac + 1 CGP: 0.78 ± 0.04, *p* < 0.0001). Moreover, compared with the TBI + NS group, Iba-1 expression levels were significantly lower in all baclofen-treated groups except in the antagonist group (TBI + 1 mg/kg baclofen + 1 mg/kg CGP35348, TBI + 1 bac + 1 CGP group) (TBI + 0.05 bac: *p* = 0.004; TBI + 0.2 bac: *p* = 0.003; TBI + 1 bac: *p* = 0.019; TBI + 1 bac + 1 CGP; *p* = 0.98). Notably, compared with the TBI + 1 bac + 1 CGP group, differences in Iba-1 expression levels were observed in groups that received 0.05 mg/kg and 0.2 mg/kg of baclofen (TBI + 0.05 bac: *p* = 0.004; TBI + 0.2 bac: *p* = 0.003; TBI + 1 bac; *p* = 0.124).

**Figure 3:**
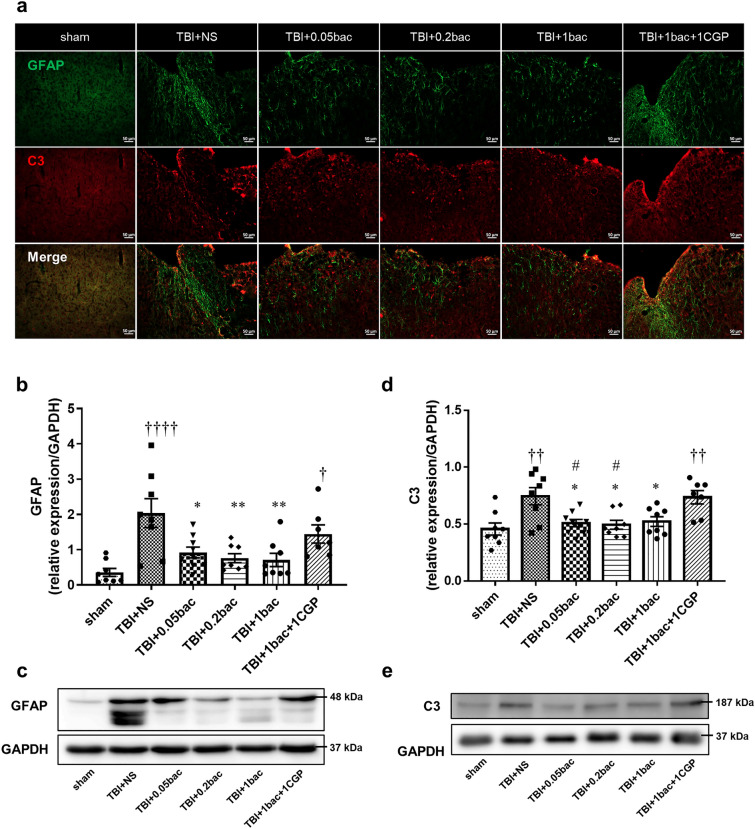
Activated astrocytes and complement C3 expression were downregulated by baclofen. (**a**) Brain tissues from the TBI + NS, TBI + bac, and TBI + 1 bac + 1 CGP treatment groups were co-stained with GFAP (green) and the complement C3 (red), 6 weeks after TBI. The above images show the cortex region. Scale bar: 50 μm. (**b**–**e**) Analysis of western blot was also conducted from cortex areas to confirm the protein levels of GFAP and C3; quantified values are shown with bar graphs and band images. Data from bar graphs are represented as the mean ± SEM (n = 7–9). One-way ANOVA with Tukey’s multiple comparison test was performed. † *p* < 0.05, †† *p* < 0.01; compared with sham. ** *p* < 0.01 compared with TBI + 0.05 bac.

To investigate whether baclofen reduces proinflammatory cytokines induced by injured microglia, the protein level of IL-1β was verified via western blot analysis (Fig. 3d,e). A significant upregulation of IL-1β expression was observed in the TBI and TBI + 1 bac + 1 CGP groups compared with the sham (sham: 0.57 ± 0.04; TBI + NS: 1 ± 0.14, *p* = 0.02; TBI + 0.05 bac: 0.7 ± 0.09, *p* = 0.89; TBI + 0.2 bac: 0.51 ± 0.05, *p* = 0.99; TBI + 1 bac: 0.53 ± 0.04, *p* = 0.99; TBI + 1 bac + 1 CGP: 1.08 ± 0.15, *p* = 0.005). When comparing among the TBI-induced groups, a significant reduction in IL-1β expression was observed in those treated with 0.02 mg/kg and 1 mg/kg of baclofen compared with the TBI + NS group (TBI + 0.05 bac: *p* = 0.2; TBI + 0.2 bac: *p* = 0.006; TBI + 1 bac: *p* = 0.009; TBI + 1 bac + 1 CGP; *p* = 0.98). Furthermore, a significant decrease in IL-1β expression levels was observed in the groups treated with 0.02 mg/kg and 1 mg/kg of baclofen compared with in the TBI + 1 bac + 1 CGP group (TBI + 0.05 bac: *p* = 0.059; TBI + 0.2 bac; *p* = 0.001; TBI + 1 bac: *p* = 0.002). These findings underscored the potential of baclofen administration, particularly at a dose of 0.02 mg/kg, in mitigating microglial activation and IL-1β expression following TBI. Full blots were shown in supplementary information (Figure S1).

### Baclofen suppressed TBI-induced activated astrocyte and related immune molecule

To confirm the degree of activation of astrocytes and immune components that exerts neurotoxicity around the peripheral cortex, GFAP and complement C3 were stained with immunofluorescence 6 weeks after TBI (Fig. 3a). The result indicated that GFAP around the peri-lesion cortex of the TBI + NS group was overly expressed compared with the sham group. Moreover, the expression of C3 colocalized with GFAP increased 6 weeks after controlled cortical impact (CCI) modeling (Figure S2). Immunoblotting was conducted to quantify GFAP protein levels in the perilesional cortex (Fig. 3b,c), revealing that GFAP expression level was significantly upregulated in the TBI + NS group and TBI + 1 bac + 1 CGP than in the sham group (sham: 0.36 ± 0.11; TBI + NS: 2.04 ± 0.41, *p* < 0.0001; TBI + 1bac + 1CGP: 1.44 ± 0.26, *p* = 0.025). Compared with the TBI + NS group, intensities of GFAP were attenuated in all doses of baclofen groups (TBI + 0.05 bac: 0.92 ± 0.15,* p* = 0.01; TBI + 0.2 bac: 0.76 ± 0.13, *p* = 0.003; TBI + 1 bac: 0.71 ± 0.19, *p* = 0.002). However, no significant difference was observed between the antagonist and baclofen groups (TBI + 0.05 bac:* p* = 0.59; TBI + 0.2 bac: *p* = 0.33; TBI + 1 bac: *p* = 0.26).

In addition, we quantified the complement C3 using western blotting (Fig. 3d,e). The C3 protein levels in the TBI + NS group and TBI + 1 bac + 1 CGP group were significantly higher than that in the sham group (sham: 0.46 ± 0.05; TBI + NS: 0.74 ± 0.08, *p* = 0.003; TBI + 1bac + 1CGP: 0.74 ± 0.06, *p* = 0.007). Meanwhile, C3 expressions were significantly downregulated at all doses of baclofen compared with the TBI + NS group (TBI + 0.05 bac: 0.5 ± 0.03,* p* = 0.02; TBI + 0.2 bac: 0.49 ± 0.04, *p* = 0.015; TBI + 1 bac: 0.52 ± 0.04, *p* = 0.04). Compared with the antagonist group, significant difference was shown in groups receiving 0.05 mg/kg and 0.2 mg/kg but not 1 mg/kg of baclofen (TBI + 0.05 bac:* p* = 0.03; TBI + 0.2 bac: *p* = 0.02; TBI + 1 bac: *p* = 0.06). These findings implied that baclofen treatment may reduce the upregulated astrocyte and toxic factor after TBI. The supplementary information included full blots (Figure S3).

### Baclofen reduced apoptotic cells in the peri-lesion cortex after TBI

**Figure 4:**
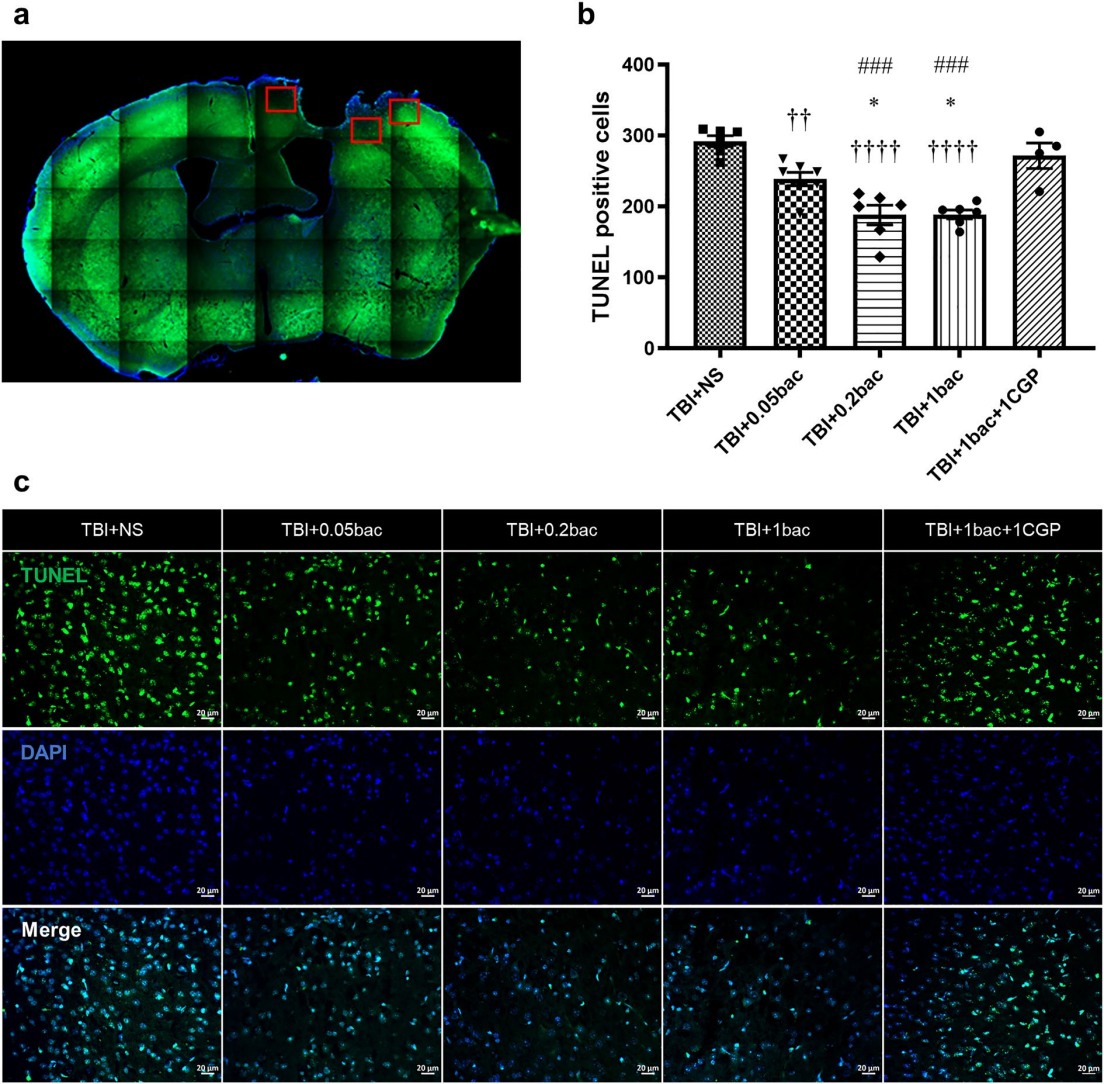
Baclofen reduced the number of apoptotic cells induced by TBI. (**a**) A schematic drawing indicates quantified areas in the cerebral cortex. (**b**) The bar graph displays the average number of TUNEL-positive cells in the peri-lesion cortex at 3 different locations, 6 weeks after TBI. Data are represented as the mean ± SEM (n = 4–7). One-way ANOVA with Tukey’s multiple comparison test was performed. †† *p* < 0.01, †††† *p* < 0.0001; compared with sham. * *p* < 0.05; compared with TBI + NS. ### *p* < 0.0005; compared with TBI + 1 bac + 1 CGP. (**c**) The fluorescence staining images of TUNEL (green) and DAPI (blue) in the cortex area. Scale bar: 20 μm.

Neuronal cell death was evaluated 6 weeks after trauma induction using TUNEL staining in injured brain tissues. For the analysis, we counted three random areas of the peripheral lesion cortex using Image J (Fig. 4a). We observed that the number of TUNEL-positive cells was significantly reduced in the baclofen-treated group than in the TBI + NS group (Fig. 4b; TBI + NS: 292 ± 7.64; TBI + 0.05 bac: 238.7 ± 9.57, *p* = 0.0095; TBI + 0.2 bac: 188 ± 13.83, *p* < 0.0001; TBI + 1 bac: 188.8 ± 6.33,* p* < 0.0001). We also discovered that the number of dead cells was significantly lower in the TBI + 0.2bac and TBI + 1bac groups than in the TBI + 0.05bac group (TBI + 0.2bac: *p* = 0.014, TBI + 1bac:* p* = 0.016). Furthermore, the antagonist group exhibited a significant increase in the number of dead cells when compared with the groups receiving 0.2 mg/kg and 1 mg/kg of baclofen (TBI + 1bac + 1CGP: 271 ± 17.95, TBI + 0.2bac: *p* = 0.0004, TBI + 1bac:* p* = 0.0005). Representative TUNEL images of each group are shown in Fig. 4c.

### Baclofen rescued dysfunction of neurological behavior and cognition

**Figure 5:**
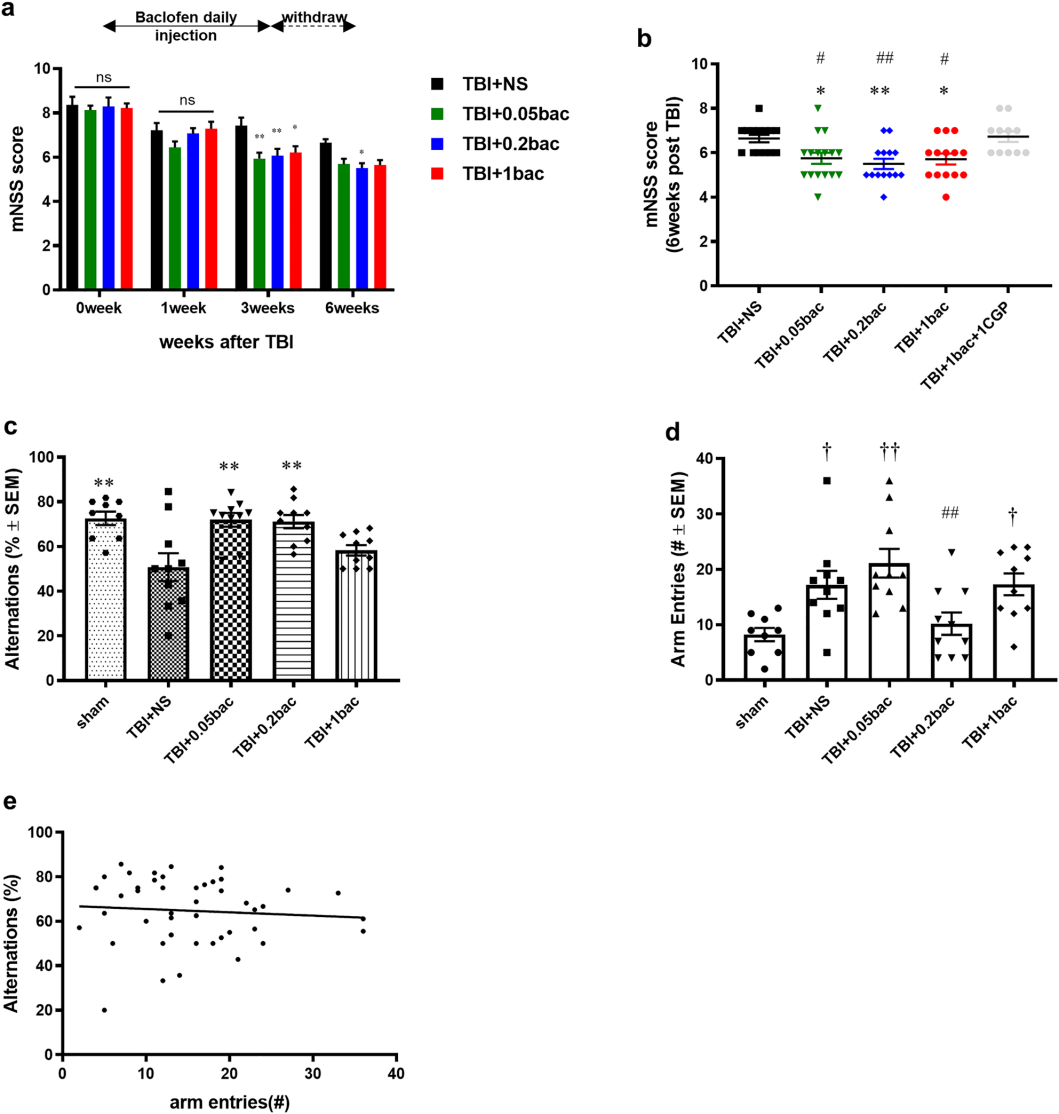
Acutely administered baclofen diminished the impairment of motor and cognitive function until the chronic phase. (**a**) mNSS was scored at 0, 1, 3, and 6 weeks after TBI in the sham (n = 9), TBI + NS (n = 14), TBI + 0.05 bac (n = 16), TBI + 0.2 bac (n = 14) and TBI + 1 bac (n = 14) groups. Data are presented as the mean ± SEM followed by two-way ANOVA with Tukey’s multiple comparison test. * p < 0.05, ** p < 0.01; compared with TBI + NS. (**b**) Analysis and comparison of mNSS data 6 weeks among the groups. One-way ANOVA was conducted with Tukey’s multiple comparison test. * p < 0.05, ** p < 0.01; compared with TBI + NS. #* p* < 0.05, ##* p* < 0.01; compared with TBI + 1 bac + 1 CGP. (**c**) Mice (n = 9–10 in each group) were randomly chosen and underwent the Y-maze test 6 weeks after TBI. One-way ANOVA was conducted with Tukey’s multiple comparison test. ** p < 0.01; compared with TBI + NS. (**d**) The number of arm entries was counted to confirm mice locomotion. One-way ANOVA was conducted with Tukey’s multiple comparison test. ** p < 0.01; compared with TBI + NS. (**e**) Pearson correlation between alternation rate and the number of arm entries was performed to verify whether motility affected voluntary movement (r =  − 0.08).

Behavioral test was conducted with modified neurological severity score (mNSS) to record neurological dysfunction, including beam, balance, and sensation. All mice behaviors were measured at 0, 1, 3, and 6 weeks after TBI modeling (Fig. 5a). The result demonstrated that scores in the baclofen groups were significantly lower than that in the TBI + NS group (7.43 ± 0.36) at 3 weeks post-(TBI + 0.05 bac: 5.94 ± 0.27, *p* = 0.0012; TBI + 0.2 bac: 6.07 ± 0.3, *p* = 0.0055; TBI + 1 bac: 6.21 ± 0.28, *p* = 0.0166). After drug withdrawal, the last test was conducted 6 weeks after TBI, revealing that only the TBI + 0.2 bac group (5.5 ± 0.23, *p* = 0.0279) showed a significantly decreased score compared with the TBI + NS group (6.64 ± 0.17; TBI + 0.05 bac: 5.94 ± 0.27, *p* = 0.0012; TBI + 1 bac; 6.21 ± 0.28, *p* = 0.0166). When comparing the scores only on the last day (Fig. 5b), the baclofen-treated group showed significant decrease compared with TBI + NS group (TBI + 0.05 bac: *p* = 0.04, TBI + 0.2 bac: *p* = 0.006, TBI + 1 bac: *p* = 0.04), whereas the neurological recovery score increased when the GABA-B receptor antagonist (6.64 ± 017. *p* = 0.999 compared with the TBI + NS) was co-administered to the mice. Furthermore, we performed an analysis with respect to the presence of the baclofen antagonist and the results were similar to those when compared with the TBI + NS group (TBI + 0.05 bac: *p* = 0.037, TBI + 0.2 bac: *p* = 0.006, TBI + 1 bac: p = 0.035). This observation revealed the potential of baclofen to ameliorate neurological dysfunction.

We further investigated the Y-maze test to measure spatial memory in 9–10 TBI mice randomly assigned to each group 6 weeks after TBI induction (Fig. 5c). Spontaneous alternation was defined as the number of consecutive three arms for total entries. The alternation rate was significantly decreased in the TBI + NS group (50.8 ± 6.27, *p* = 0.0023) than in the sham group (72.61 ± 2.97), indicating that brain trauma worsened working memory. Contrastingly, the alternation rate increased in the TBI + 0.05 bac (71.17 ± 2.96, *p* = 0.0024) and TBI + 0.2 bac groups (71.17 ± 2.96, *p* = 0.0037) than in the TBI + NS group, whereas no change was observed in the TBI + 1 bac group (58.3 ± 2.31, *p* = 0.62). These results indicates that a low dose of early baclofen improved cognitive function, whereas a relatively high dose deteriorate cognitive function. Moreover, no correlation was observed between the alternation rate and number of arm entries, implying that locomotor activity did not affect spontaneous alternations (Fig. 5d,e).

## Discussion

Neuroinflammation is a contributing factor to secondary injury that induces a poorer long-term prognosis for patients with TBI^22,23^. In the acute TBI phase, neuroinflammation displays a neuroprotective property by anti-inflammatory mediators to restore the disrupted blood–brain barrier (BBB); however, the sustained proliferation of microglia and astrocytes can convert to noxious action in the chronic stage after TBI^6,24^. This sustained deterioration eventually contributes to chronic degeneration such as dementia or encephalopathy^25,26^. Considering this transition time gap, previous studies have speculated that early medical intervention in the recovery phase may effectively prevent secondary injury from TBI^27,28^.

**Figure 6:**
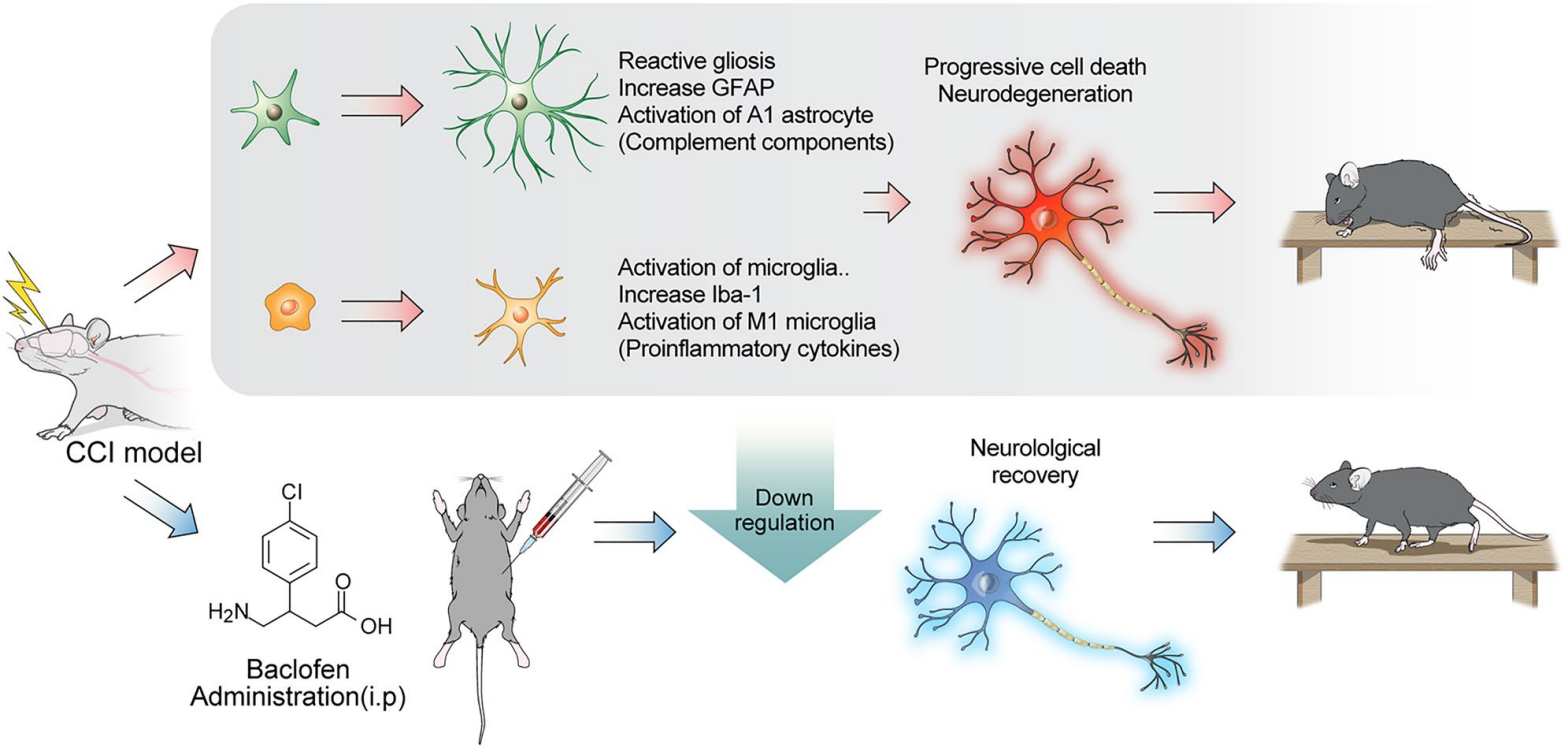
Graphical summary of the experiment. Reactive glial activation is a major cascade of neuroinflammation in TBI. Prolonged M1 subtype of microglia and A1 of astrocytes activation initiates release of neurotoxic immune molecules, with progressive cell death leading to neurodegeneration. Administered baclofen in the early TBI phase can downregulate continuous neuroinflammation with enhanced neurological behaviors even in chronic periods.

Currently, baclofen, a selective GABA-B receptor agonist, has demonstrated unexpected but beneficial effects on patients or animal models with CNS disorders^29–32^. The original purpose of baclofen was to treat spasticity by reducing the muscle tone of patients with motor function loss^29,33,34^. However, based on the evidence that GABA-B receptors are predominantly expressed in neurons, microglia, and astrocytes, increasing investigations have shown that baclofen modulates activated immune cells, particularly in neurodegenerative diseases^35,36^. However, research on the anti-inflammatory effects of baclofen after TBI remains unclear. Thus, we mainly focused on mitigating the activation of primary immune cells in the relatively late stage of TBI after early continuous baclofen administration. Herein, we proved that early administration of baclofen impeded the over-activation of microglia, astrocytes and related immune molecules and recovered neurobehavioral dysfunction in TBI mice. Hence, acute intervention with a relatively low-dose baclofen can be a potential treatment for secondary damage caused by TBI (Fig. 6).

In this study, we first confirmed that early intervention of baclofen mitigated the extent of lesion tissue compared with the TBI group (Fig. 2). These outcomes may have effectively prevented further tissue loss by attenuating the excessive activation of microglia and astrocytes^37–39^. After glial activation, IL-1β, known to be released from harmful microglia, was strongly expressed around the acute to the chronic TBI phase with neuronal degeneration^40,41^. Upregulated A1 astrocytes also initiate the neurotoxic inflammatory complement pathway with high levels of C3 after traumatic injury^42,43^. Particularly, the cell density of activated Iba-1 and GFAP was highly measured in the cortex up to 1 year following injury, causing continuous damage^38,39^. However, we prove that acute to subacute treatment with baclofen significantly downregulated microglia and IL-1β expression in the injured cortex at 6 weeks post-TBI (Fig. 2b,d). In addition, baclofen reduced the overexpression of GFAP and C3 protein levels in the same region (Fig. 3b,d). Microglia and astrocyte downregulation appeared to have resulted in fewer apoptotic cells in the lesion of treated group (Fig. 5). Previous studies confirmed that baclofen reduced TNF-α and IL-1β expression in the MPTP-induced Parkinson’s disease rat model^18^. In other preclinical studies, baclofen also decreased Iba-1 expression in spinal cord injury and GFAP expression in AD^21,32^. These results are similar to our findings, suggesting baclofen can be used as early intervention in patients with TBI. Particularly, the group that received a combination of baclofen and its antagonist (CGP35348) exhibited similar Iba-1 and GFAP expression level to those of the TBI group. These findings implied that baclofen may directly suppress the activity of major deleterious immune molecules.

However, the mechanism underlying the protective effects of baclofen remains unclear. One plausible mechanism is that GABA-B receptors are involved in the enhancement of receptor recycling. The expression of GABA-B receptors on the cell surface is accurately regulated through a balance of recycling, degradation, and delivery of synthesized receptors^44^. GABA-B receptors exhibit constitutive internalization and are then recycled to the cell surface or degraded in lysosomes^45^. However, under excitotoxic conditions, GABA-B receptors were significantly reduced. A study by Hlehil showed that continuous stimulation of GABA-B receptors by baclofen in cerebral ischemia models could normalize cell surface GABA-B receptor expression levels through a fast recycling pathway, leading to decreased neuronal excitability and processive loss of neurons^31^. Some researchers also suggest that the activation of GABA-B receptor supports neuronal survival via the PI3K/Akt-GSK3β pathway^46–48^. Under chronic cerebral hypoperfusion, chronic administration of baclofen notably alleviated neuronal damage through upregulating Bcl-2/Bax ratio and increasing Akt, GSK-3β, and ERK activation, which suppressed detrimental cellular autophagy^48^. These mechanisms are expected to contribute to the protective effects of baclofen. However, as this study did not investigate the underlying mechanisms of baclofen, further research on this topic is required^47,48^.

As an extension of histological findings, we investigated the outcomes of baclofen’s treatment with behavioral tests. First, we performed the mNSS tests to validate whether baclofen rescues neurological dysfunction (Fig. 5a,b). Notably, the score was not decreased at immediate drug injection but significantly reduced in the baclofen groups at 3 weeks after drug administration with dose-inverse relationship. The effect was most significantly maintained at a dose of 0.2 mg/kg, suggesting that repetitive administration of baclofen is more effective than short-term treatment, at least for moderate-severe TBI. However, during the Y-maze test, we observed that memory function was enhanced in an inverse relationship to baclofen concentration; 0.05 mg/kg and 0.2 mg/kg of baclofen, which were relatively low doses in this study, significantly improved cognition in TBI, whereas the 1 mg/kg appeared to deteriorate memory function (Fig. 5c). This may be because excessive GABA-B receptors may disrupt the cholinergic or glutamatergic system, which plays a critical role in learning and memory^49,50^. Consistent with our results, previous studies have shown that low doses of baclofen improve cognitive function, where relatively higher doses do not. Levin et al. reported a deleterious effect on memory function at 0.5 mg/kg and 1 mg/kg of baclofen administration, whereas in normal rats receiving 0.125 mg/kg and 0.25 mg/kg, the baclofen induced-memory impairment was reversed, as observed in the radial arm maze^51^. Moreover, Pilipenko et al. showed that 0.025 mg/kg and 0.05 mg/kg of baclofen decreased memory impairment in an AD rat model in the water maze test^21^. However, the correlation between anti-neuroinflammation and cognitive improvement was limited, particularly that associated with baclofen. Thus, further studies identifying the factors related to plasticity are necessary to determine the exact mechanism of baclofen.

Collectively, mNss tests showed the potential anti-neuroinflammatory effect of baclofen. Despite the 20-fold difference between the minimum (0.05 mg/kg) and maximum doses (1 mg/kg), no significant differences were observed among 0.05, 0.2 and 1 mg/kg doses. However, the Y-maze test results suggest that continuous 1 mg/kg baclofen administration may have an adverse effect on memory function^52,53^. Therefore, optimizing dosage within the lower dose range appears to be more beneficial. However, a report showed that baclofen faces challenges in crossing the BBB and requires higher doses to achieve therapeutic levels^54^. Thus, further studies should be conducted to find the optimal dosage for diverse routes.

Although we observed the effectiveness of baclofen in mitigating the inflammatory response, the absence of precise dosage criteria caused us to conduct experiments using doses of 0.05, 0.2, and 1 mg/kg. For clinical use, baclofen is administered intrathecally to TBI patients at a concentration of approximately 50 mcg. In our animal model, administering the drug daily via a pump was challenging; thus, we opted for intraperitoneal injection. In clinical setting, the initial drug concentration serves as a reference point, and the dosage varies depending on individual patient responsiveness. For instance, a concentration of 1400 mcg can be utilized, which, when converted to the intraperitoneal route, corresponds to 220 mg. Therefore, the intraperitoneal concentration of baclofen employed in this study is not significantly different from the doses typically delivered via intrathecal pumps. Our findings are meaningful because the acute therapeutic effects were retained until the late phase despite drug withdrawal, and the study was designed considering clinically applicable time points and doses of baclofen treatment. Nevertheless, further investigations on acute medical applicability and pharmacological dynamics of baclofen in TBI are required to affirm the neuroprotective role of baclofen^55^.

This study has some limitations. First, in this study, CCI mice were established to imitate the pathological consequences of moderate-to-severe TBI^56,57^. The injury levels and outcomes could vary by species or between patients. Hence, our results should be interpreted with caution when considering the clinical application of baclofen. While our model may not be directly transferable to a broader TBI patient population, it shows the possibility, particularly within the scope of the TBI parameters explored in this study. Second, we evaluate the therapeutic efficacy of baclofen by specifically targeting the lesion. However, long-term adverse effects of TBI can extend to the white matters of the brain or even beyond the ipsilateral hemisphere, encompassing the contralateral hemisphere and other surrounding areas^58–60^. Particularly, some researchers observed the accumulation of tau protein in the contralateral hippocampus 6 weeks after TBI occurrence^58^. This extensive damage may potentially contribute to cognitive decline in TBI-induced long-term damage^61–63^. Nevertheless, the primary objective of our study was to ascertain whether baclofen demonstrates therapeutic efficacy in a TBI model. Hence, the verification of its effects primarily focused on the lesion. We believe that future studies can yield more comprehensive results by conducting analyses of the entire brain, by conducting an analysis related to the synaptic plasticity. Finally, we conducted the study using only male mice. Previous reports have indicated that hormonal fluctuations in female subjects can impact certain behavioral experiments^64^. However, it is essential to underscore that, in the context of treatment effectiveness, sex is not expected to play a significant role in the overall activation.

## Conclusions

Early administration of baclofen exerted a neuroprotective effect in TBI models by suppressing hyper-activated gliosis and immune factors, regardless of dose. Baclofen recovered sensorimotor functions and cognition at a lower dose; therefore, early intervention with low baclofen treatment can be considered an adequate therapeutic approach. Although we did not investigate the detailed mechanism of how baclofen regulates the immune system in this study, the findings provide evidence that baclofen can be a therapeutic option for TBI patients who suffer from spasticity and secondary injury.

## Materials and methods

### Animals

**Figure 7:**
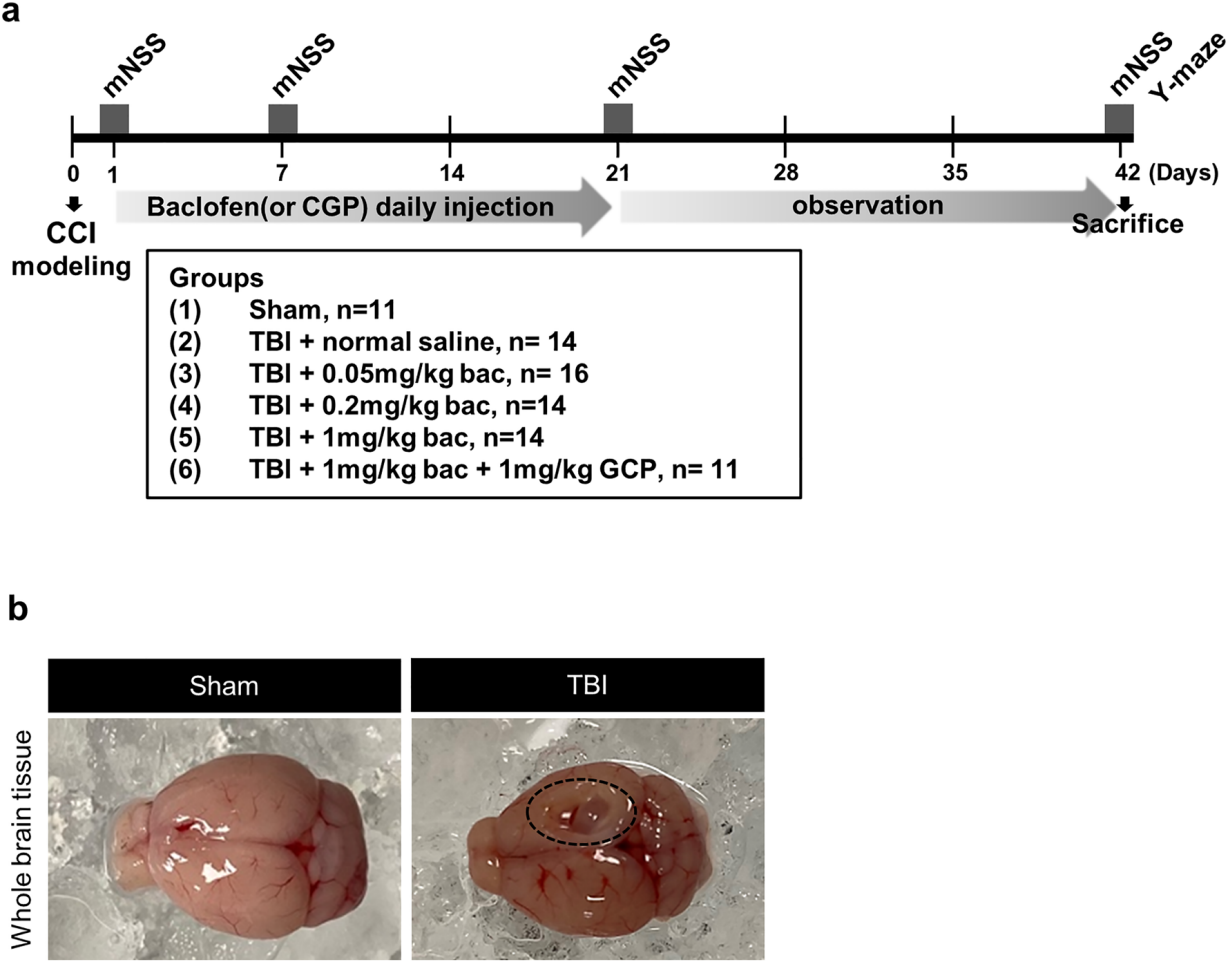
Scheme of the experimental procedure and image of TBI whole brain tissue. (**a**) Twenty-four h after TBI induction, mice were randomized to receive baclofen (or baclofen with CGP) intraperitoneally or in an equal volume of normal saline for 3 weeks. The mNSS test was performed four times, and the Y-maze test was performed once on the last day of observation. (**b**) In whole brain tissue images, a part of the sensorimotor cortex region disappeared at 6 weeks post-injury, compared with the sham group. All histological investigations were verified in the perilesional cortex (black dotted line).

Male C57BL/6 mice (aged 10–12 weeks, weight 20–25 g) were used in this study (Table 1). They were housed in groups of five per cage under a temperature (of 23 ± 2 °C) and an automatic 12 h light/dark cycle with food and water. The mice were randomly assigned to the following groups; sham (n = 11), TBI + normal saline (NS) (n = 14), TBI + 0.05 mg/kg baclofen (n = 16), TBI + 0.2 mg/kg baclofen (n = 14), TBI + 1 mg/kg baclofen (n = 14), and TBI + 1 mg/kg baclofen + 1 mg/kg CGP35348 (n = 11). The mice were then coded using alphanumeric labels for each subject, and data analysis was conducted by researchers who were blinded to the group identities. The experimental time flow is shown in Fig. 7a. This study was conducted in accordance with the Guide for the Care and Use of Laboratory Animals of the National Institutes of Health. All animal experiments were approved by the Institutional Animal Care and Use Committee of Yonsei University (IACUC number: 2021–0296) and in compliance with the animal Research: Reporting of In Vivo Experiments (ARRIVE) guidelines.

**Table 1:** Number of animals per group used for each analysis.

Groups	For western blot	For IHC	Total
Sham	8	3	11
TBI + NS	8	6	14
TBI + 0.05 mg/kg	9	7	16
TBI + 0.2 mg/kg	8	6	14
TBI + 1 mg/kg	8	6	14
TBI + 1 mg/kg + 1 CGP	7	4	11

### Controlled cortical impact model for TBI mice

Male C57BL/6 mice were anesthetized with 1–1.5% isoflurane and fixed in the stereotaxic frame of the impact device PSI-0310 (Precision Systems and Instrumentation, USA). The right hemisphere of the sensorimotor cortex and hippocampus was targeted, where AP was -1 mm and ML was 2 mm to the bregma. An incision was made on the scalp, and a 3 mm diameter craniotomy was performed with a drill. The impact parameter was decided to make moderate to severe TBI; velocity was 5 m/s, the dwelling time was 100 ms, and lesion depth was 1 mm. After the damage was done to the brain, the mice were sutured and kept in a warm blanket until they awakened from the anesthetic. The same surgery was performed in the sham group; however, there was no impact (Fig. 7b).

### Baclofen administration and sacrifice

NS and baclofen were administered by intraperitoneal injection (i.p.) for 3 consecutive weeks a day after TBI. Baclofen was purchased from Sigma Aldrich, UK (B5399) and dissolved in NS at a concentration of 0.05 mg/kg, 0.2 mg/kg, and 1 mg/kg. CGP35348 was obtained from Tocris, UK (1245) and dissolved in the same solvent. Baclofen concentrations were determined with reference to the clinical dosage. Mice were administered baclofen a day after TBI induction for 3 consecutive weeks. After injections were completed, the drug was withdrawn from the mice, and they underwent additional observation periods for 3 weeks. They were then sacrificed at 6 weeks after CCI modeling.

### Assessment of neurological injury

To evaluate the degree of neurological impairment induced by TBI, the mNSS was recorded. The task includes the beam walking test (0.5, 1, 2, 3 cm in width), balance test (0.5, 0.75, 1 cm in diameter), straight walk, seeking behavior, and others, and all these sums make the total score of 12 points. Mice performed the test at 0, 1, 3, and 6 weeks after TBI induction and were awarded one point when they failed the task.

### The Y-maze test

Spontaneous alternation in the Y-maze was performed 6 weeks after TBI induction to confirm memory impairment. The alternation task was tested using a symmetrical Y-maze apparatus consisting of three equal arms (length: 40 cm, height: 15 cm, width: 9 cm) made of black acrylic plastic. All mice were placed in the center of the maze and allowed to explore for 8 min. All movements were recorded with a video camera, and the alternation rate was analyzed by the number of correct alternations/number of total arm entries × 100.

### Toluidine blue staining for lesion size measurement

Toluidine blue staining was performed to assess extent in lesion size in mice brains 6 weeks after TBI modeling. Frozen sectioned tissues were washed three times with 1X PBS. They were then mounted on glass slides and rinsed with a toluidine blue working solution for 10 s. The slides were washed three times with distilled water and quickly dehydrated with alcohol. The stained tissues were then covered with a glass cover slip. The slides were observed using a microscope at 1.25 × magnification. Percentage of the lesion size = (contralateral cortex area − ipsilateral spare cortex area)/(contralateral cortex area) × 100%. The quantification was performed using MATLAB.

### Immunofluorescence (IF)

Animals were anesthetized with a ketamine cocktail (60 mg/kg, i.p.) and perfused with NS and 4% paraformaldehyde. The brains were then cut into 15 or 30 μm coronal sections using a freezing microtome (Leica Biosystems, Germany) and stored in a cryoprotectant solution consisting of 30% sucrose, 1% polyvinylpyrrolidone (Sigma-Aldrich), and 30% ethylene glycol (Thermo Fisher Scientific, USA) in PBS at -20 °C. Fluorescence immunohistochemistry was performed to detect microglia, astrocytes, and complement C3. Sections were washed with PBS and blocked with 5% normal goat serum (Vector Laboratories, USA) and then incubated with primary antibodies at the following dilutions: Iba-1 (019–19,747; 1:300; Wako Chemicals, USA), GFAP (ab4674; 1:400; Abcam, U.K.), and complement C3 (ab200999; 1:400; Abcam, U.K.). After the primary immunoreaction, the sections were incubated with secondary antibodies conjugated with Alexa Fluor 633 (A20991; 1:200; Thermo Fisher Scientific) or Alexa Fluor 488 (A11001; 1:400; Thermo Fisher Scientific). The staining intensity of the sections was visualized using an M2 microscope (Carl Zeiss, Germany).

### Western blot analysis

Western blot samples were prepared on the same day as the IF sample without perfusion and isolated from the peripheral cortex. They were homogenized in a lysis buffer (PRO-PREP; Intron Biotechnology) and placed on ice for 30 min, followed by centrifugation at 13,000 rpm for 30 min; the protein concentration in the lysate was measured using the bicinchoninic acid-protein assay reagent kit (#23,227, Thermo Fisher Scientific). Next, 15 or 20 μg of each protein sample was size-separated using sodium dodecyl sulfate–polyacrylamide gel electrophoresis and transferred to a polyvinylidene fluoride membrane using a Bio-Rad Trans-Blot apparatus. The membranes were incubated with a blocking buffer containing 5% non-fat dry milk in TBST for 1 h at room temperature. The membranes were then incubated with primary antibodies overnight at 4 °C, with rabbit anti-Iba1 (ab178846; 1:1000; Abcam), rabbit anti-IL-1β (ab9722; 1:1000; Abcam), rabbit anti-GFAP (ab7260; 1:5000; Abcam), rabbit anti-C3(ab200999; 1:1000; Abcam), and rabbit anti-GAPDH (#2118, 1:2000, Cell Signaling Technology). The membranes were washed three times for 5 min each with TBST and incubated with corresponding secondary antibodies, with goat anti-rabbit IgG(H + L)-HRP (GenDEPOT, USA) for 2 h at room temperature. Immunoreactive bands were visualized using an enhanced chemiluminescent solution (West Save Gold, AbFrontier Inc.) and developed with Amersham ImageQuant 800 Western blot imaging station (Cytiva). The intensity of each band was determined using an analysis system (Multi Gauge version 3.0; Fujifilm).

### Labeling of apoptotic cells

The TUNEL (terminal deoxynucleotidyl transferase-mediated dUTP nick-end labeling) assay was performed using the ApopTag Plus Fluorescein In Situ Apoptosis Detection kit (#S7111, Millipore) following the indicated protocol. Briefly, the tissues were fixed in 1% paraformaldehyde and washed twice in PBS. They were post-fixed in precooled ethanol and acetic acid solution for 5 min at − 20 °C and incubated with an equilibration buffer for 10 min. After that, the tissues were incubated with the terminal deoxynucleotidyl transferase at 37 °C for 1 h, washed with stop/wash buffer for 10 min, and then incubated with anti-dioxygenin conjugate for 30 min at room temperature. The tissues were washed with PBS, and DAPI was applied (D1306; 1:1000, Invitrogen) for 30 min. The last wash was done four times, and the tissues were covered with a mounting medium under a glass coverslip. The slides were viewed using a fluorescein microscope.

### Statistical analysis

All results are expressed as the mean ± standard error of the mean (SEM) and were analyzed using GraphPad Prism 8 (GraphPad Software, USA). All data were passed D'Agostino & Pearson or Shapiro–Wilk normality test. Histological data were analyzed using one-way analysis of variance (ANOVA) followed by Tukey’s post hoc test. One-way or two-way ANOVA was performed for the behavioral test. Statistical significance was considered at *p* < 0.05. The Pearson correlation test was also performed to show the statistical relationship between the two variables.

### Supplementary Information

Supplementary Figures.

## Data Availability

The datasets generated during and/or analyzed during the current study are available from the corresponding author on reasonable request.

## References

[1] Werner C, Engelhard K (2007). Pathophysiology of traumatic brain injury. Br. J. Anaesth..

[2] Greve MW, Zink BJ (2009). Pathophysiology of traumatic brain injury. Mt. Sinai J. Med..

[3] Crupi R, Cordaro M, Cuzzocrea S, Impellizzeri D (2020). Management of traumatic brain injury: From present to future. Antioxidants (Basel).

[4] Dinet V, Petry KG, Badaut J (2019). Brain-immune interactions and neuroinflammation after traumatic brain injury. Front. Neurosci..

[5] Sulhan S, Lyon KA, Shapiro LA, Huang JH (2020). Neuroinflammation and blood-brain barrier disruption following traumatic brain injury: Pathophysiology and potential therapeutic targets. J. Neurosci. Res..

[6] Karve IP, Taylor JM, Crack PJ (2016). The contribution of astrocytes and microglia to traumatic brain injury. Br. J. Pharmacol..

[7] Jassam YN, Izzy S, Whalen M, McGavern DB, El Khoury J (2017). Neuroimmunology of traumatic brain injury: Time for a paradigm shift. Neuron.

[8] Menon DK (2009). Unique challenges in clinical trials in traumatic brain injury. Crit. Care Med..

[9] Loane DJ, Faden AI (2010). Neuroprotection for traumatic brain injury: Translational challenges and emerging therapeutic strategies. Trends Pharmacol. Sci..

[10] van Erp IAM (2023). Tackling neuroinflammation after traumatic brain injury: Complement inhibition as a therapy for secondary injury. Neurotherapeutics.

[11] Pérez-Arredondo A (2016). Baclofen in the therapeutic of sequele of traumatic brain injury: Spasticity. Clin. Neuropharmacol..

[12] Ertzgaard P, Campo C, Calabrese A (2017). Efficacy and safety of oral baclofen in the management of spasticity: A rationale for intrathecal baclofen. J. Rehabil. Med..

[13] Al-Khodairy AT, Wicky G, Nicolo D, Vuadens P (2015). Influence of intrathecal baclofen on the level of consciousness and mental functions after extremely severe traumatic brain injury: Brief report. Brain Inj..

[14] Halbmayer LM (2022). On the recovery of disorders of consciousness under intrathecal baclofen administration for severe spasticity-An observational study. Brain Behav..

[15] Stetkarova I, Kramska L, Keller J (2021). Improvement of memory functions in chronic spinal cord injury after long-term intrathecal baclofen delivery for spasticity relief. Neuromodulation.

[16] Brown KM, Roy KK, Hockerman GH, Doerksen RJ, Colby DA (2015). Activation of the γ-aminobutyric acid type B (GABA(B)) receptor by agonists and positive allosteric modulators. J. Med. Chem..

[17] Crowley T, Cryan JF, Downer EJ, O’Leary OF (2016). Inhibiting neuroinflammation: The role and therapeutic potential of GABA in neuro-immune interactions. Brain Behav. Immun..

[18] Tyagi RK (2015). Possible role of GABA-B receptor modulation in MPTP induced Parkinson’s disease in rats. Exp. Toxicol. Pathol..

[19] Liu F (2019). GABA(B) receptor activation attenuates inflammatory orofacial pain by modulating interleukin-1beta in satellite glial cells: Role of NF-kappaB and MAPK signaling pathways. Brain Res. Bull..

[20] Crowley T (2015). Modulation of TLR3/TLR4 inflammatory signaling by the GABAB receptor agonist baclofen in glia and immune cells: Relevance to therapeutic effects in multiple sclerosis. Front. Cell Neurosci..

[21] Pilipenko V (2018). Very low doses of muscimol and baclofen ameliorate cognitive deficits and regulate protein expression in the brain of a rat model of streptozocin-induced Alzheimer’s disease. Eur. J. Pharmacol..

[22] Schimmel SJ, Acosta S, Lozano D (2017). Neuroinflammation in traumatic brain injury: A chronic response to an acute injury. Brain Circ..

[23] Lassaren P (2021). Systemic inflammation alters the neuroinflammatory response: A prospective clinical trial in traumatic brain injury. J Neuroinflammation.

[24] Simon DW (2017). The far-reaching scope of neuroinflammation after traumatic brain injury. Nat. Rev. Neurol..

[25] van Erp IAM (2022). Tackling neuroinflammation after traumatic brain injury: Complement inhibition as a therapy for secondary injury. Neurotherapeutics.

[26] Cherry JD (2016). Microglial neuroinflammation contributes to tau accumulation in chronic traumatic encephalopathy. Acta Neuropathol. Commun..

[27] Prabhakar NK, Khan H, Grewal AK, Singh TG (2022). Intervention of neuroinflammation in the traumatic brain injury trajectory: In vivo and clinical approaches. Int. Immunopharmacol..

[28] Doganyigit Z (2022). The role of neuroinflammatory mediators in the pathogenesis of traumatic brain injury: A narrative review. ACS Chem. Neurosci..

[29] Margetis K (2014). Intrathecal baclofen associated with improvement of consciousness disorders in spasticity patients. Neuromodulation.

[30] Kim W, Seo H (2014). Baclofen, a GABAB receptor agonist, enhances ubiquitin-proteasome system functioning and neuronal survival in Huntington’s disease model mice. Biochem. Biophys. Res. Commun..

[31] Hleihil M, Vaas M, Bhat MA, Balakrishnan K, Benke D (2021). Sustained baclofen-induced activation of GABA (B) receptors after cerebral ischemia restores receptor expression and function and limits progressing loss of neurons. Front. Mol. Neurosci..

[32] de Sousa N (2023). Acute baclofen administration promotes functional recovery after spinal cord injury. Spine J..

[33] Hoarau X, Richer E, Dehail P, Cuny E (2012). A 10-year follow-up study of patients with severe traumatic brain injury and dysautonomia treated with intrathecal baclofen therapy. Brain Inj..

[34] Taira T (2009). Intrathecal administration of GABA agonists in the vegetative state. Prog. Brain Res..

[35] Charles KJ, Deuchars J, Davies CH, Pangalos MN (2003). GABA B receptor subunit expression in glia. Mol. Cell Neurosci..

[36] Kuhn SA (2004). Microglia express GABA(B) receptors to modulate interleukin release. Mol. Cell Neurosci..

[37] Hanell A, Hedin J, Clausen F, Marklund N (2012). Facilitated assessment of tissue loss following traumatic brain injury. Front. Neurol..

[38] Loane DJ, Kumar A, Stoica BA, Cabatbat R, Faden AI (2014). Progressive neurodegeneration after experimental brain trauma: Association with chronic microglial activation. J. Neuropathol. Exp. Neurol..

[39] Sofroniew MV (2009). Molecular dissection of reactive astrogliosis and glial scar formation. Trends Neurosci..

[40] Semple BD (2017). Interleukin-1 receptor in seizure susceptibility after traumatic injury to the pediatric brain. J. Neurosci..

[41] Li Q, Zhang H, Liu X (2022). Didymin alleviates cerebral ischemia-reperfusion injury by activating the PPAR signaling pathway. Yonsei Med. J..

[42] Pekna M, Pekny M (2021). The complement system: A powerful modulator and effector of astrocyte function in the healthy and diseased central nervous system. Cells.

[43] Alawieh A, Langley EF, Weber S, Adkins D, Tomlinson S (2018). Identifying the role of complement in triggering neuroinflammation after traumatic brain injury. J. Neurosci..

[44] Benke D (2010). Mechanisms of GABAB receptor exocytosis, endocytosis, and degradation. Adv. Pharmacol..

[45] Grampp T, Sauter K, Markovic B, Benke D (2007). Gamma-aminobutyric acid type B receptors are constitutively internalized via the clathrin-dependent pathway and targeted to lysosomes for degradation. J. Biol. Chem..

[46] Tu H (2010). GABAB receptor activation protects neurons from apoptosis via IGF-1 receptor transactivation. J. Neurosci..

[47] Sun Z, Sun L, Tu L (2020). GABAB receptor-mediated PI3K/Akt signaling pathway alleviates oxidative stress and neuronal cell injury in a rat model of Alzheimer’s disease. J. Alzheimers Dis..

[48] Liu L (2015). Baclofen mediates neuroprotection on hippocampal CA1 pyramidal cells through the regulation of autophagy under chronic cerebral hypoperfusion. Sci. Rep. U.K..

[49] Nakagawa Y, Ishibashi Y, Yoshii T, Tagashira E (1995). Involvement of cholinergic systems in the deficit of place learning in Morris water maze task induced by baclofen in rats. Brain Res..

[50] Izquierdo I, Medina JH (1995). Correlation between the pharmacology of long-term potentiation and the pharmacology of memory. Neurobiol. Learn. Mem..

[51] Levin ED, Weber E, Icenogle L (2004). Baclofen interactions with nicotine in rats: Effects on memory. Pharmacol. Biochem. Behav..

[52] Holajova M, Franek M (2018). Effect of short- and long-term administration of baclofen on spatial learning and memory in rats. Physiol. Res..

[53] Zarrindast MR, Khodjastehfar E, Oryan S, Torkaman-Boutorabi A (2001). Baclofen-impairment of memory retention in rats: Possible interaction with adrenoceptor mechanism(s). Eur. J. Pharmacol..

[54] Heetla HW, Proost JH, Molmans BH, Staal MJ, van Laar T (2016). A pharmacokinetic-pharmacodynamic model for intrathecal baclofen in patients with severe spasticity. Br. J. Clin. Pharmacol..

[55] Al Shoyaib A, Archie SR, Karamyan VT (2020). Intraperitoneal route of drug administration: Should it be used in experimental animal studies?. Pharm. Res. Dordr..

[56] Siebold L, Obenaus A, Goyal R (2018). Criteria to define mild, moderate, and severe traumatic brain injury in the mouse controlled cortical impact model. Exp. Neurol..

[57] Chiu CC (2016). Neuroinflammation in animal models of traumatic brain injury. J. Neurosci. Methods.

[58] Zhao ZA (2017). Perivascular AQP4 dysregulation in the hippocampal CA1 area after traumatic brain injury is alleviated by adenosine A2A receptor inactivation. Sci. Rep. U.K..

[59] Johnson VE (2013). Inflammation and white matter degeneration persist for years after a single traumatic brain injury. Brain.

[60] McDonald S, Dalton KI, Rushby JA, Landin-Romero R (2019). Loss of white matter connections after severe traumatic brain injury (TBI) and its relationship to social cognition. Brain Imaging Behav..

[61] Laurent C (2017). Hippocampal T cell infiltration promotes neuroinflammation and cognitive decline in a mouse model of tauopathy. Brain.

[62] Piirainen S (2017). Psychosocial stress on neuroinflammation and cognitive dysfunctions in Alzheimer’s disease: The emerging role for microglia?. Neurosci. Biobehav. Rev..

[63] Chun MY (2022). (18)F-THK5351 PET positivity and longitudinal changes in cognitive function in beta-amyloid-negative amnestic mild cognitive impairment. Yonsei Med. J..

[64] Ogawa S, Chan J, Gustafsson JÅ, Korach KS, Pfaff DW (2003). Estrogen increases locomotor activity in mice through estrogen receptor α: Specificity for the type of activity. Endocrinology.

