# Supplementary material for: Long-term results on the suppression of secondary brain injury by early administered low-dose baclofen in a traumatic brain injury mouse model
Source: Sci Rep. 2023 Oct 30;13:18563. doi: 10.1038/s41598-023-45600-7 (PMC10616194; doi:10.1038/s41598-023-45600-7)

Supplementary information

Long-term results on the suppression of secondary brain injury by early administered low-dose baclofen in a traumatic brain injury mouse model

Ji Young Park[1†], Junwon Park[1†], Jiwon Baek[1], Jin Woo Chang[1,3], Young Goo Kim[2*], and Won Seok Chang[1*]

[1]Department of Neurosurgery, Yonsei University College of Medicine, Seoul, Korea

[2]Department of Neurosurgery, Ewha Womans University School of Medicine, Ewha Womans University Mokdong Hospital, Seoul, Korea

[3]Brain Korea 21 PLUS Project for Medical Science & Brain Research Institute, Yonsei University College of Medicine, Seoul, Korea

† Ji Young Park and Junwon Park contributed equally to this work as co-first authors.

* Young Goo Kim and Won Seok Chang contributed equally to this work as co-corresponding authors.

*Corresponding authors

Won Seok Chang, M.D., Ph.D. E-mail: CHANGWS0716@yuhs.ac

Young Goo Kim, M.D., E-mail: ygkim@ewha.ac.kr

Figure S1. Uncropped western blot bands of Iba-1 and IL-1β with GAPDH.

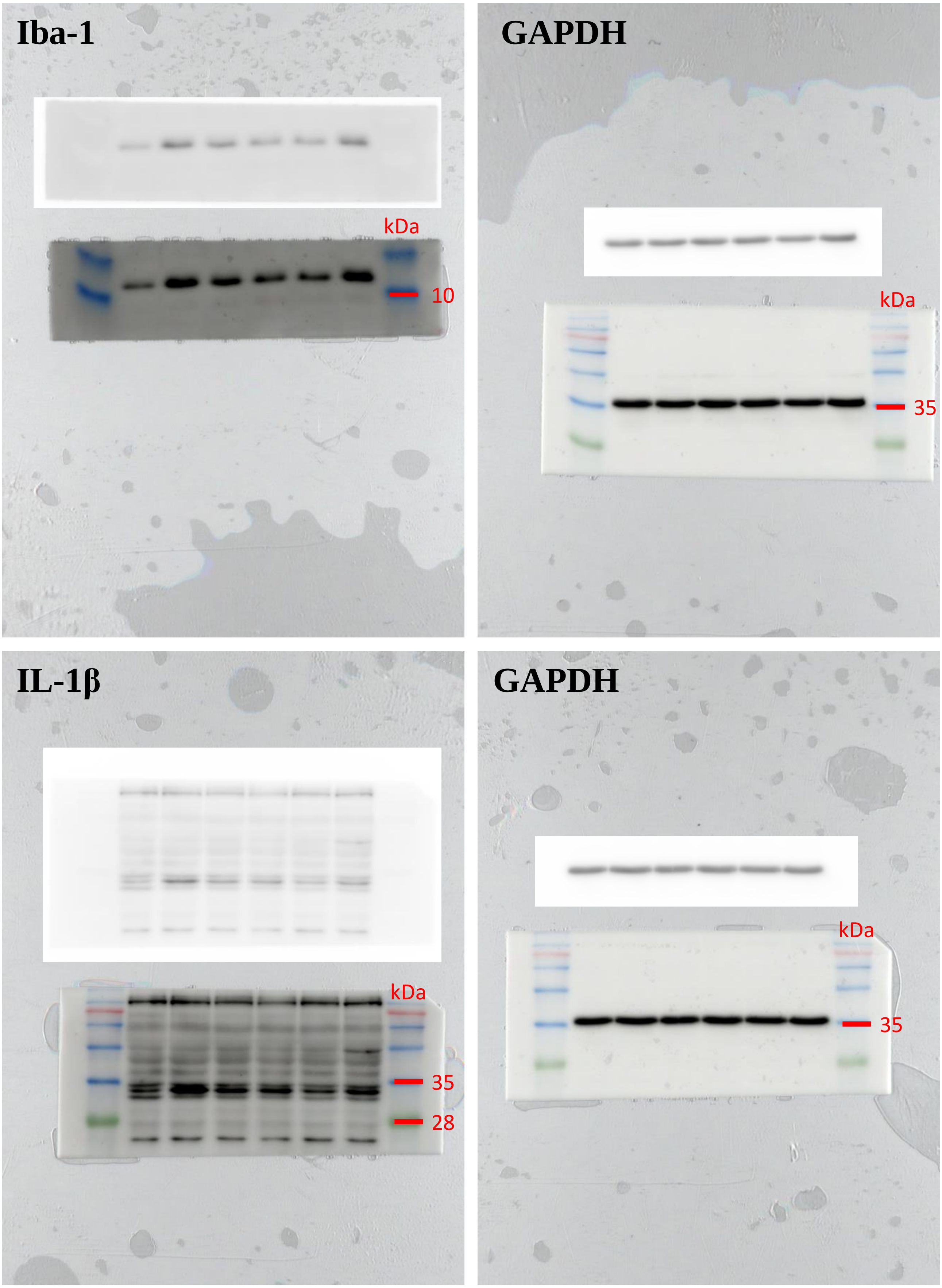

Figure S2. The total number of C3 and co-localized number with astrocytes after TBI.

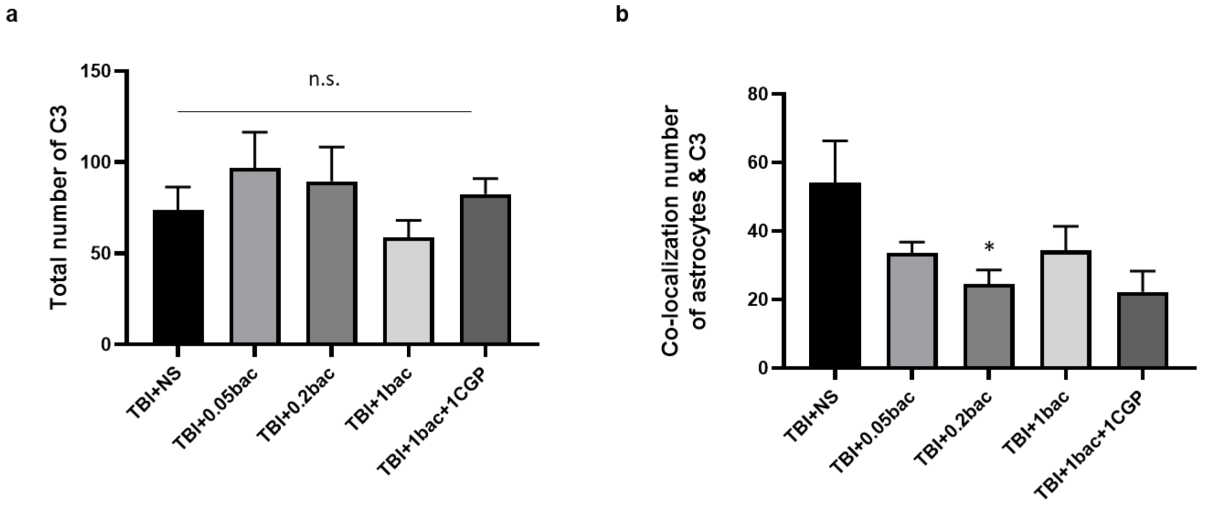

(a) The total number of C3 counted in IHC images. All data are represented as the mean±SEM (n=4-7). One-way ANOVA with Tukey's multiple comparison test was performed. There were no significance among groups ($p>0.5$ when using ANOVA) (b) The number of C3 that co-localized with astrocytes. All data are represented as the mean±SEM (n=4-7). One-way ANOVA with Tukey's multiple comparison test was performed. * $p<0.05$ compared with TBI+NS.

Figure S3. Uncropped western blot bands of GFAP and complement C3 with GAPDH

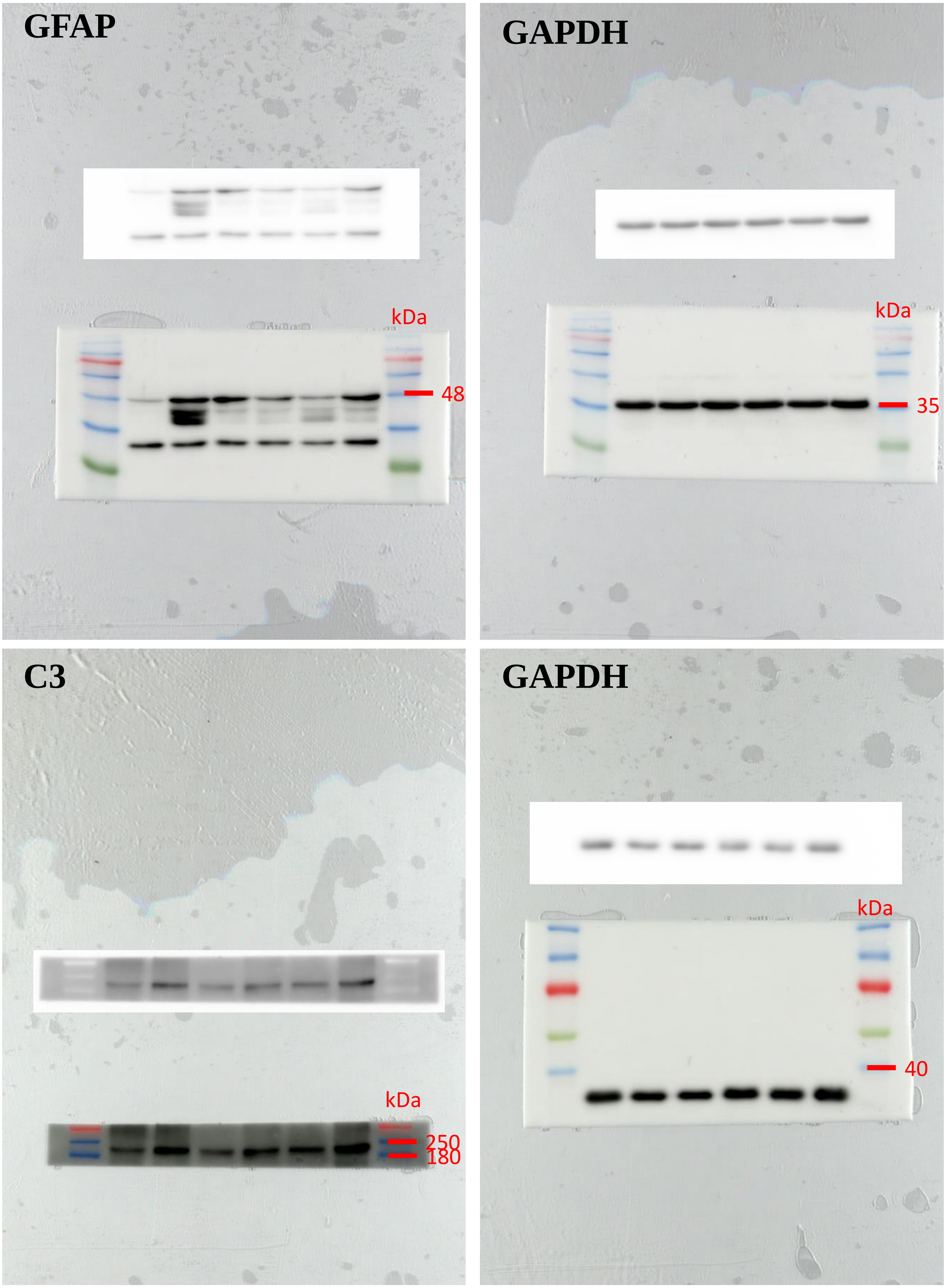

Supplement: Supplementary file 1 — Supplementary Figures. [file 41598_2023_45600_MOESM1_ESM.pdf]
